# Supplementary material for: Identification of photoperiod-induced specific miRNAs in the adrenal glands of Sunite sheep (Ovis aries)
Source: Front Vet Sci. 2022 Jul 22;9:888207. doi: 10.3389/fvets.2022.888207 (PMC9354845; doi:10.3389/fvets.2022.888207)
Supplement: Supplementary file 1 [file Data_Sheet_1.ZIP › Supplementary file/miRDeep2 file.zip!/miRDeep2 file/Know_Structure/SPLP42A3/oar-mir-494.pdf]

miRBase precursor : oar-mir-494
Total read count : 7854
oar-miR-494-5p read count : 31
oar-miR-494-3p read count : 7823
remaining reads : 0

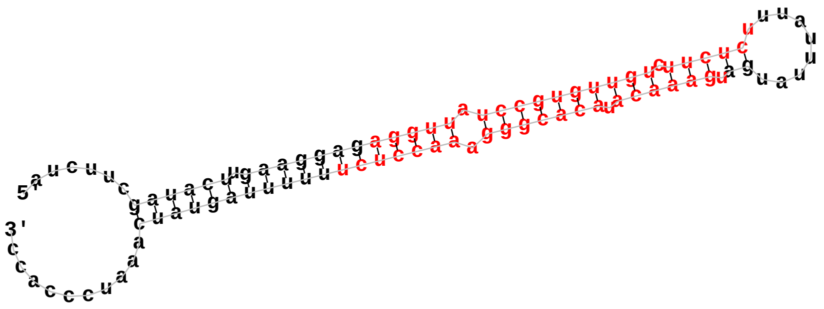

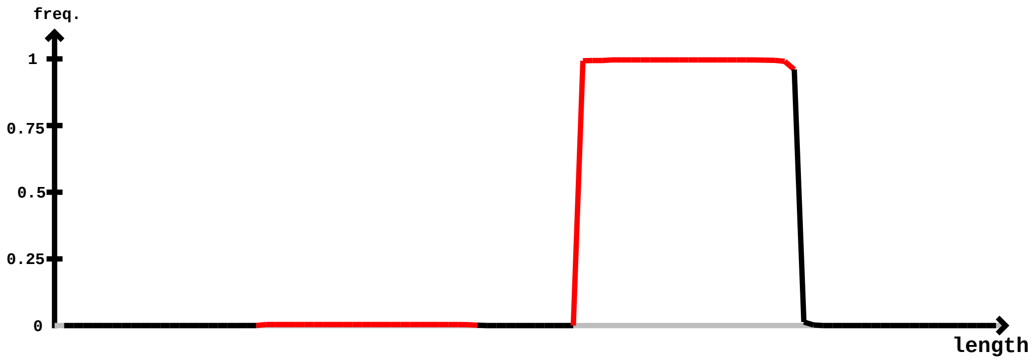

oar-miR-494-3p

oar-miR-494-5p

```
5'- aucuucgauacuugaaggagagguuauccguguugucuucucuuuauuuaugaugaaacauacacgggaaaccucuuuuuuaguaucaaaucccacc  -3'   exp
    ......(((((.(((((((((((.((((((((((.(((((.........)).)))))).))))))).)))))))))))))))).........   reads   mm   sample
    ..................agagguuauccguguugucuu..........................................................   1   0   seq
    ....................agguuauccguguugucuucu........................................................   4   0   seq
    ....................agguuauccguguugucuucuc.......................................................   15   0   seq
    ....................agguuauccguguugucuucucu......................................................   11   0   seq
    .....................................................ugaaacauacacgggaaa..........................   1   0   seq
    .....................................................ugaaacauacacgggaaac.........................   6   0   seq
    .....................................................ugaaacauacacgggaaacc........................   6   0   seq
    .....................................................ugaaacauacacgggaaaccu.......................   29   0   seq
    .....................................................ugaaacauacacgggaaaccuc......................   239   0   seq
    .....................................................ugaaacauacacgggaaaccucu.....................   7431   0   seq
    .....................................................ugaaacauacacgggaaaccucuu....................   86   0   seq
    .....................................................ugaaacauacacgggaaaccucuuu...................   1   0   seq
    ......................................................gaaacauacacgggaaaccucu.....................   6   0   seq
    ........................................................aacauacacgggaaaccucuu....................   2   0   seq
    ........................................................aacauacacgggaaaccucuuu...................   16   0   seq
```
